# Hospital discharge abstracts have limited accuracy in identifying occurrence of Clostridium difficile infections among hospitalized individuals with inflammatory bowel disease: A population-based study

**DOI:** 10.1371/journal.pone.0171266

**Published:** 2017-02-15

**Authors:** Harminder Singh, Zoann Nugent, B. Nancy Yu, Lisa M. Lix, Laura Targownik, Charles Bernstein

**Affiliations:** 1 Internal Medicine, University of Manitoba, Winnipeg, Manitoba, Canada; 2 University of Manitoba IBD Clinical and Research Center, Winnipeg, Manitoba, Canada; 3 Community Health Sciences, University of Manitoba, Winnipeg, Manitoba, Canada; 4 CancerCare Manitoba, Department of Epidemiology and Cancer Registry, Winnipeg, Manitoba, Canada; 5 Public Health Branch, Manitoba Health, Seniors and Active Living, Winnipeg, Manitoba, Canada; Cleveland Clinic, UNITED STATES

## Abstract

**Background:**

Hospital discharge databases are used to study the epidemiology of Clostridium difficile infections (CDI) among hospitalized patients with inflammatory bowel disease (IBD). CDI in IBD is increasingly important and accurately estimating its occurrence is critical in understanding its comorbidity. There are limited data on the reliability of the International Classification of Diseases 10th revision (ICD-10) (now widely used in North America) CDI code in determining occurrence of CDI among hospitalized patients. We compared the performance of ICD-10 CDI coding to laboratory confirmed CDI diagnoses.

**Methods:**

The University of Manitoba IBD Epidemiology Database was used to identify individuals with and without IBD discharged with CDI diagnoses between 07/01/2005 and 3/31/2014. Sensitivity, specificity, positive predictive value (PPV) and negative predictive value (NPV) of ICD-10 CDI code was compared to laboratory CDI diagnoses recorded in a province wide CDI dataset. Multivariable logistic regression models were performed to test the predictors of diagnostic inaccuracy of ICD-10 CDI code.

**Results:**

There were 273 episodes of laboratory confirmed CDI (hospitalized and non-hospitalized) among 7396 individuals with IBD and 536 among 66,297 matched controls. The sensitivity, specificity, PPV and NPV of ICD-10 CDI code in discharge abstracts was 72.8%, 99.6%, 64.1% and 99.7% among those with IBD and 70.8%, 99.9%, 79.0% and 99.9% among those without IBD. Predictors of diagnostic inaccuracy included IBD, older age, increased co-morbidity and earlier years of hospitalization.

**Conclusions:**

Identification of CDI using ICD-10 CDI code in hospital discharge abstracts may not identify up to 30% of CDI cases, with worse performance among those with IBD.

## Introduction

Clostridium difficile is the most commonly reported pathogen causing healthcare associated infections and can lead to clinically significant diarrhea and substantial morbidity and mortality[1]. Clostridium difficile infection (CDI) can often occur in people with pre-existing inflammatory bowel disease (IBD). The occurrence of CDI concomitantly with IBD has been associated with increased length of hospital stay, increased colectomy rate and higher mortality when compared with hospitalized IBD patients without CDI[2]. However, much of the information on the epidemiology of CDI among persons with IBD comes from an assessment of hospital discharge databases, using the International Classification of Diseases, 9^th^ revision, Clinical Modification (ICD-9-CM) code for CD (008.45)[3–5]. Use of coding in the hospital discharge databases eliminates the need to obtain data directly from multiple diagnostic laboratories in a large jurisdiction and has facilitated the surveillance of CDI. However, the accuracy of using hospital discharge abstracts ICD 9 codes to accurately identify CDI in the general population has been variable and to the best of our knowledge never evaluated among those with IBD[6,7].

International Classification of Diseases, 10th revision (ICD-10) coding has been implemented in Canada over the last decade and is currently being introduced in the US. The ICD-10 system is a more specific coding system in general and could lead to improvement in coding and ascertainment of various medical conditions in hospital discharge abstracts. A recent study involving tertiary care hospitals in Calgary, Canada reported excellent performance of ICD-10 code A04.7 among patients with ulcerative colitis(UC) with sensitivity, specificity, positive predictive value (PPV), and negative predictive value (NPV) of 82.1% (95% Confidence Interval (CI): 71.7–89.9%), 99.4% (95% CI: 99.1–99.7%), 88.4% (95% CI: 82.9–92.3%), and 99.1% (95% CI: 98.5–99.4%), respectively[8]. Comparison to performance among individuals without IBD was not performed, which is important to assess the effect of using ICD-10 CDI codes on differences in incidence and outcomes to that among individuals without IBD. An earlier study from France did report marked underestimation of CDIs when using ICD-10 code A04.7 among the general population[9]. If the results among those with IBD in Calgary could be generalized to IBD in other settings and jurisdictions this would facilitate the evaluation of CDI in IBD in various settings as the hospital discharge databases are now widely available.

We therefore performed a population based study to determine the test performance characteristics of ICD-10 code A04.7 in hospital discharge abstracts to identify CDIs among individuals with IBD and matched controls without IBD, in comparison to laboratory confirmed CDI diagnoses in an entire province.

## Methods

### Data sources

Manitoba is a central Canadian province with a relatively stable population of approximately 1.3 million according to the 2011 Statistics Canada Census. Manitoba Health, Seniors and Active Living (MH), a publicly funded agency of the Government of Manitoba provides comprehensive universal health insurance to all Manitoba residents. MH maintains several centralized and electronic administrative databases, including the MH Population Registry, hospital discharge, physician claims and prescription dispensation records. Several prior studies have validated the accuracy of these data[10,11]. The MH Population Registry is a database for all residents in the province and is used by MH to track eligibility for provincial health care insurance coverage. The Physician Claims database contains information for each physician service, including the patients’ identification, date of service, diagnosis (three digit ICD-9 CM codes) and service tariff code. The Hospital Discharge Abstract Database (DAD) includes for each hospitalization, the patient’s identification, dates of admission and discharge, details of attending physicians, up to 25 ICD-10 diagnoses and 20 procedures performed during the hospitalization (Manitoba hospitals switched to ICD-10 and Canadian Classification of Interventions (CCI) on April1, 2004). A unique personal health identification number (PHIN), assigned to all Manitobans since 1984, can be used to link the patient records in various databases.

The University of Manitoba IBD Epidemiology Database (UMIBDED) was initiated in 1995 and is recurrently updated using MH administrative databases and therefore contains all of the information listed above for MH administrative databases[12]. The case definition of IBD in UMIBDED includes individuals with at least 5 separate physician contacts and/or hospitalizations for an IBD diagnosis (≥ 3 contacts for those residing in Manitoba for ≤ 2 years). This case definition has been previously validated, with a sensitivity and specificity of approximately 90% in comparison with both patient self-report and chart review[12]. The specificity of 90% refers to the specificity among those with at least one physician or hospital claim for diagnosis of IBD. Since the majority of residents of Manitoba have no claims for IBD, the specificity of this definition is close to 100% in the general population. Individuals are identified to have Crohn's disease (CD) or UC based on the majority of their last 9 claims. The UMIBDED has been used for many epidemiological studies[13–17]. Each individual with IBD in the UMIBDED is matched to 10 randomly selected individuals without IBD based on age, sex and postal area of residence on the date of IBD diagnosis (index date). The index date is assigned by the date of IBD diagnosis, defined as the date for the first claim for IBD, which also serves as the index date for their matches. All study subjects have to be registered with MH and living in Manitoba on the index date. The IBD incident date is assigned as the date for the first claim for IBD for individuals with first claim in 1987 or later and a minimum of 3 year prior lead in time period of residence in the province.

The MH Public Health Branch Epidemiology and Surveillance has maintained a population-based CDI dataset since 2005, developed from the legally mandated universal reporting of CDI cases in the province- a copy of all positive reports is sent by the reporting laboratories to the Surveillance unit. The MH CDI dataset includes identifying information on the individuals with CDI, including their PHIN, postal code of residence at time of CDI diagnosis, the date the stool specimen was collected and results reported. Between 2005 and May 2013, the laboratories in Manitoba used as a first step in CDI testing, immunoassays for the Glutamate Dehydrogenase antigen (GD antigen) and CDI toxins antigen tests, followed by the cytopathic effect (CPE) assay and/or culture for discordant results. Immunoassay for GDH has been reported to have high negative predictive value and that for CDI toxins low sensitivity; however the algorithm used has been previously reported in a Manitoba study to have PPV as well as NPV of over 95%[18]. Since May 2013, the Nucleic Acid Amplification Test (NAAT) is used for confirmation for faster turnaround time[18]. Diagnostic testing for CDI is performed in six public laboratories, of which 3 perform most of the testing. Only loose stool, which takes shape of its container have been tested by the laboratories, thereby minimizing the detection of asymptomatic carriers. The UMIBDED and the MH CDI dataset were linked for the current study using scrambled anonymized PHINs.

### Study cohort

All individuals with IBD and their matched controls who were residents of Manitoba between July 1 2005 and March 31, 2014 were identified from the UMIBDED and included in the study if they had an overnight hospitalisation for any reason at any hospital in the province during this time period.

### Study measures and outcomes

The CDIs were defined as laboratory confirmed CDI from the MH CDI dataset (gold standard); those with specimen collection between July 1, 2005 and March 31, 2014 were included in the study. Socioeconomic status (SES) was designated using the Socioeconomic Factor Index (SEFI), a previously developed and validated measure, which is based on several neighborhood level social determinants of wealth[19]. The Charlson co-morbidity index (CCI) was used to categorise the comorbidities, determined from ambulatory care visits and inpatient hospitalisations in the year preceding the index hospitalisation. Hospitals in Winnipeg, the largest city in the province with two-thirds of the provincial population, were considered urban and all other hospitals rural.

### Statistical analysis

Using the MH CDI database as the gold standard, the sensitivity, specificity, PPV and NPV and corresponding 95% CIs of the ICD-10 code A04.7 in the DAD were calculated. Fisher’s exact test was used to compare test performance between individuals with and without IBD and between individuals with a diagnosis of CD vs. a diagnosis of UC. In the primary analysis, only specimens collected during the hospital admission were included. We hypothesized that the DAD may include CDI diagnosed from specimens collected immediately prior to the index hospitalization and hence analyses were also performed for specimens collected in the index hospital stay or the prior 2 weeks; and for specimens collected or reported during the index hospital stay. Youden's J statistic (also called Youden's index)[20], a measure of the overall diagnostic test accuracy, was calculated.

Multivariable logistic regression models were used to test the potential explanatory variables associated with false negatives (FNs), false positives (FPs) and diagnostic inaccuracy (FNs plus FPs) of the ICD-10 code as compared with lab diagnosis of CDIs. Specimens collected during index hospitalisations and the preceding two weeks were included in this potential predictor analysis as the diagnostic accuracy (Youden's index) for the ICD-10 CDI code among individuals with IBD was the highest for the specimens collected in this time period. Variables assessed included patient demographics (age at index hospital admission, sex), diagnosis of IBD, CCI score, SEFI, hospital characteristic (urban vs rural) and year of hospitalization (categorised in 3 time periods/era-2005-07; 2008–10; 2011–2014). The Brier score, a measure of the accuracy of probabilistic predictions was calculated for the models-the value of Brier score ranges between 0 and 1, with lower scores interpreted as more accurate[21]. The c-statistic (equivalent to the area under the Receiver Operating Characteristic curve for binary outcomes), a measure of the discriminative performance of the logistic models, was also calculated[22].

For the instances of FP and FN ICD-10 CDI code, we assessed for CDI diagnoses in the MH CDI laboratory dataset prior to and after the index hospitalization to assess whether reporting outside of the hospitalization time period was a reason for FPs and FNs.

This study was approved by the University of Manitoba’s Health Research Ethics Board and MH’s Health Information and Privacy Committee. The ethics committee waived the need for consent from study subjects.

## Results

A total of 29, 554 individuals with 69,645 admissions were included in the study. Of 7396 individuals with IBD (52% UC, 48% CD) there were 13, 139 hospitalizations and of 66,297 without IBD there were 56,506 hospitalizations between July 1, 2005 and March 30, 2014. There were 809 episodes of CDI (hospitalized and non-hospitalized) after the IBD diagnosis/index date (matching date) among the study cohorts in this time period (CD: 115; UC: 158; controls: 536).

When only the specimens collected during the index hospitalizations were included in the definition of a gold standard laboratory diagnosis of CDI, the overall sensitivity of ICD-10 CDI code in DAD among the study cohorts was 71.4% (95% CI: 67.0–75.9%) and PPV was 73.6% (95% CI: 69.3–77.9%) with a significantly lower PPV of 64.1% (95% CI: 56.3–71.8%) among those with IBD Table 1. The sensitivity and PPV were higher among those with IBD at 73.8% (95% CI: 66.4–80.5%) and 77.5% (95% CI: 70.4–83.8%), respectively, when specimens collected during the index hospitalization or the preceding two weeks were included in the definition of the “gold standard” CDI. However, irrespective of the definitions used, PPV of ICD-10 code A04.7 for CDI was lower among those with IBD. There was no significant difference in performance of ICD-10 CDI code in the DAD among those with UC vs. those with CD.

**Table 1:** Performance of ICD-10 Clostridium Difficile code A047 in hospital discharge abstracts in comparison with the laboratory diagnosis of clostridium difficile, by timing of collection of samples.

Study group	Total number of hospitalizations	TP (n)	FP (n)	FN (n)	Sensitivity (%) (95% CI)	Specificity (%) (95% CI)	PPV (%) (95% CI)	NPV (%) (95% CI)	Youden’s J(%)
Sample collected during index hospitalization									
All	69645	290	104	116	71.4 (67.0–75.9)	99.8 (99.8–99.9)	73.6 (69.3–77.9)	99.8 (99.8–99.9)	71
No IBD	56506	199	53	82	70.8 (65.5–76.2)	99.9 (99.9–99.9)	79.0 (73.8–83.7)	99.9 (99.8–99.9)	71
IBD	13139	91	51	34	72.8 (64.8–80.4)	99.6 (99.5–99.7)	64.1 (56.3–71.8)	99.7 (99.7–99.8)	72
*P value comparing IBD vs*. *no IBD*					0.72	<0.0001	0.002	0.006	
Crohns	7412	43	20	19	69.4 (58.1–80.6)	99.7 (99.6–99.8)	68.3 (57.1–79.4)	99.7 (99.6–99.9)	69
UC	5727	44	31	15	76.2 (65.1–85.7)	99.5 (99.3–99.6)	60.8 (49.4–70.9)	99.7 (99.6–99.9)	76
*P value comparing Crohns vs*. *UC*					0.43	0.02	0.38	1.0	
Sample collected during hospitalization or the preceding two weeks									
All	69645	330	64	137	70.7 (66.6–74.7)	99.9 (99.9–99.9)	83.8 (79.9–87.3)	99.8 (99.8–99.8)	71
No IBD	56506	220	32	98	69.2 (64.2–74.2)	99.9 (99.9–100)	87.3 (82.9–91.3)	99.8 (99.8–99.9)	69
IBD	13139	110	32	39	73.8 (66.4–80.5)	99.8 (99.7–99.8)	77.5 (70.4–83.8)	99.7 (99.6–99.8)	74
*P value comparing IBD vs*. *no IBD*					0.33	<0.0001	0.015	0.006	
Crohns	7412	50	13	20	71.4 (60.0–81.4)	99.8 (99.7–99.9)	79.4 (68.3–88.9)	99.7 (99.6–99.8)	71
UC	5727	60	19	19	75.9 (65.8–84.8)	99.7 (99.5–99.8)	75.9 (65.8–84.8)	99.7 (99.5–99.8)	76
*P value comparing Crohns vs*. *UC*					0.58	0.07	0.69	0.52	
Sample collected or reported during hospitalization									
All	69645	312	82	121	72.1 (67.9–76.2)	99.9 (99.9–99.9)	79.2 (75.1–83.2)	99.8 (99.8–99.9)	72
No IBD	56506	213	39	84	71.7 (66.7–76.8)	99.9 (99.9–100)	84.5 (79.8–88.9)	99.9 (99.8–99.9)	72
IBD	13139	99	43	37	72.8 (65.4–80.1)	99.7 (99.6–99.8)	69.7 (62.0–77.5)	99.7 (99.6–99.8)	72
*P value comparing IBD vs*. *no IBD*					0.91	<0.0001	0.0007	0.002	
Crohns	7412	48	15	20	70.6 (58.8–80.9)	99.8 (99.7–99.9)	76.2 (65.1–85.7)	99.7 (99.6–99.8)	70
UC	5727	51	28	17	75.0 (64.7–85.3)	99.5 (99.3–99.7)	64.6 (54.4–74.7)	99.7 (99.5–99.8)	75
*P value comparing Crohns vs*. *UC*					0.70	0.005	0.15	0.87	

TP: true positive; FP: false positive; FN: false negative; PPV: positive predictive value; NPV: negative predictive value

In multivariable logistic regression models, IBD was an independent predictor of diagnostic inaccuracy of ICD-10 CDI code in the DAD (Odds Ratio (OR): 2.40; 95%CI: 1.78–3.24) Table 2. The DAD CDI diagnosis also did worse among older individuals, those with multiple co-morbidities and the earliest era of the study. The c-statistic was 0.63 to 0.67, which suggests that there are additional factors for diagnostic accuracy, which were not included in the model.

**Table 2:** Multivariable logistic regression models to assess potential predictors of overall diagnostic inaccuracy, FP and FN of ICD-10 Clostridium Difficile code A047 in comparison with laboratory diagnosis of Clostridium difficile infections.

Predictor	Overall Diagnostic Inaccuracy		FP		FN	
	OR	95% CI	OR	95% CI	OR	95% CI
IBD	**2.40**	**1.78–3.24**	**4.09**	**2.47–6.77**	**1.82**	**1.24–2.66**
No IBD	Reference					
Male	1.04	0.78–1.39	1.22	0.74–2.01	0.97	0.68–1.37
Female	Reference					
Age at index hospitalization (yrs)						
<50	**0.45**	**0.29–0.71**	0.51	0.22–1.20	**0.43**	**0.25–0.73**
50–80	**0.70**	**0.55–0.99**	0.49	0.49–1.73	**0.62**	**0.41–0.94**
>80	Reference					
CCI score						
0	Reference					
1	0.90	0.57–1.42	0.81	0.34–1.95	0.94	0.55–1.60
>1	**1.45**	**1.00–2.11**	1.71	0.87–3.33	1.35	0.86–2.12
First hospitalization	Reference					
Second or later hospitalization	**2.25**	**1.56–3.24**	**3.61**	**1.72–7.61**	**1.89**	**1.24–2.89**
Rural hospital	0.99	0.74–1.31	0.73	0.44–1.21	1.13	0.81–1.60
Urban hospital	Reference					
SEFI	0.99	0.89–1.10	1.01	0.85–1.20	0.98	0.87–1.11
Era of hospitalization						
2005–07	**1.82**	**1.28–2.58**	**1.87**	**1.04–3.38**	**1.81**	**1.18–2.79**
2008–10	1.15	0.81–1.63	0.90	0.48–1.69	1.29	0.84–1.97
2011–14	Reference					
c-statistic		0.671	0.640		0.627	
Brier Score		0.003	0.0009		0.002	

FP: false positive; FN: false negative

Of the 137 FN ICD-10 CDIs (hospitalizations median length of stay: 12 days; Interquartile range (IQR): 5–35)), 70 (56%) had CDI reported during the hospitalization, 41 (33%) had specimen collected during admission (but reported after the index hospital discharge) and 13 (10%) had specimens collected in the preceding 2 weeks.

Of the 64 FPs (hospitalizations median length of stay: 9 days; IQR: 5–23)) 36 (56%) had no record of a positive lab diagnosis of CDI, 8 (12%) had a positive specimen collected more than a month prior to admission, 8 (12%) in the 2–4 weeks before admission, 6 (9%) within a month after hospital discharge and another 6 (9%) more than a month after hospital discharge.

Since the laboratory test for CDI in Manitoba changed in May 2013, a sensitivity analysis was performed excluding the data after April 2013. There was no significant change in effect estimates. A sensitivity analysis was also performed to assess the effect of hospital size (categorized by tertiles of number of hospitalizations among the study subjects) on the test performance characteristics of ICD-10 CDI code in DAD-although the effect size estimates of test characteristics varied, the sensitivity of ICD-10 CDI code was 76% or lower in all categories and PPV of the ICD-10 CDI code among those with IBD was uniformly lower than that among those without IBD Table 3.

**Table 3:** Performance of ICD-10 Clostridium Difficile code A047 in hospital discharge abstracts in comparison with the laboratory diagnosis of clostridium difficile, by size of hospitals (for samples collected during the hospital stay).

Study group	Total number of hospitalizations	TP (n)	FP (n)	FN (n)	Sensitivity (%) (95% CI)	Specificity (%) (95% CI)	PPV (%) (95% CI)	NPV (%) (95% CI)	Youden’s J (%)
Largest hospitals (n = 2)									
All	25550	115	44	42	73.2 (66.2–80.3)	99.8 (99.8–99.9)	72.3 (65.4–79.3)	99.8 (99.8–99.9)	73
No IBD	20527	79	23	27	74.5 (66–82.1)	99.9 (99.8–99.9)	77.5 (68.6–85.3)	99.9 (99.8–99.9)	74
IBD	5023	36	21	15	70.6 (56.9–82.4)	99.6 (99.4–99.7)	63.2 (50.9–75.4)	99.7 (99.5–99.8)	70
*P value comparing IBD vs*. *no IBD*					0.71	<0.0001	0.06	0.02	
Middle sized hospitals (n = 5)									
All	22474	109	26	37	74.7 (67.1–81.5)	99.9 (99.8–99.9)	80.7 (74–87.4)	99.8 (99.8–99.9)	73
No IBD	18297	74	15	26	74.0 (65–82)	99.9 (99.9–99.9)	83.1 (75.3–91)	99.9 (99.8–99.9)	74
IBD	4177	35	11	11	76.1 (63.0–87.0)	99.7 (99.6–99.9)	76.1 (63.0–87.0)	99.7 (99.6–99.9)	70
*P value comparing IBD vs*. *no IBD*					0.84	0.004	0.36	0.09	
Smallest hospitals (n = 83)									
All	21621	66	34	37	64.1 (54.3–72.8)	99.8 (99.8–99.9)	66.0 (57.0–75.0)	99.8 (99.8–99.9)	64
No IBD	17682	46	15	29	61.3 (50.7–72)	99.9 (99.9–100)	75.4 (63.9–85.3)	99.8 (99.8–99.9)	61
IBD	3939	20	19	8	71.4 (53.6–85.7)	99.5 (99.3–99.7)	51.3 (35.9–66.7)	99.8 (99.6–99.9)	71
*P value comparing IBD vs*. *no IBD*					0.37	<0.0001	0.017	0.53	

## Discussion

In this population based study evaluating the performance of CDI ICD-10 code A047 in the hospital discharge database in comparison to the laboratory confirmed diagnosis in a cohort of IBD patients and matched non-IBD patients, we report several key findings. First we found approximately 30% of the laboratory confirmed CDI from samples collected during hospitalizations were not recorded in the hospital discharge abstracts. Second the PPV of CDI ICD-10 code A04.7 in hospital discharges database was lower among those with IBD than those without IBD. Third, there was an era effect with ICD-10 code A047 recording in DAD performing worse in the earlier years of the study. Combined, these findings suggest surveillance of CDI with hospital discharge diagnosis, should be undertaken with caution and may not provide accurate assessments of CDI among hospitalized patients, especially those with IBD.

Many surveillance studies of CDI in IBD have been performed using ICD codes, mostly ICD-9-CM code 008.45[2]. There are no validation data on use of ICD-9 CM code to identify CDI among those with IBD. However, in one large study of general population, using the ICD-9-CM code resulted in higher estimates of CDI occurrence and time trends of a greater increase in rates than that reported using the laboratory results; the use of the ICD-9-CM code overestimated the number of CDI cases relative to the use of the toxin assay[23] In that study, a total of 10,832 cases of CDI were identified of which 27% had ICD-9-CM code identification alone, 15.2% a positive toxin result only and 57.8% both. An overestimation of CDI rates using the ICD-9-CM code has been reported in other studies[24].

The ICD-10 system is a more robust and specific coding system in general and is expected to lead to improvement in coding and ascertainment of various medical conditions in hospital discharge abstracts. However, there are limited data on the performance test characteristics of using ICD-10 CDI code (A04.7) in DAD to define CDIs among hospitalized patients with IBD. The overall PPV of 73.6% in our study is very similar to the summary statistic reported for ICD-9-CM code 008.45 in a meta-analysis of US studies: 71.6% (95% CI: 62.1–86.6%)[7]. Our study findings are in contrast to those from a study of 3 large hospitals in Calgary and suggest that the use of ICD-10 CDI code may have to be validated in each setting prior to its use for estimating CDI rates and measuring outcomes in specific settings[8]. It is important to note that our study results suggest that year of hospitalization is an independent predictor of performance of the ICD-10 CDI code and hence the validation of this code in a particular year of data may not be generalizable to other years. Further, it is possible that over time the ICD-10 CDI code may have more robust reliability for identifying true CDI if for instance hospital DAD coders rely more on laboratory results for a laboratory based clinical diagnosis like CDI.

We found that one third of those with FP CDI in DAD had a positive test result specimen collected either a month prior to or after the index hospitalization. Thus the FP ICD 10 CDI coding records may be due to CDIs occurring before or after the index hospitalization or due to unconfirmed clinical suspicions. Rarely, a specimen (including a swab) cannot be obtained from a case with CDI.

It is important to note that the CDI code in the DAD did worse among individuals with IBD and older individuals. Such individuals often have other causes of diarrhea. It is likely many such individuals were clinically suspected to have CDI which was not confirmed on laboratory testing. Irrespective of the reasons for worse performance, our study highlights the serious limitations of using hospital discharge records to discern differences in CDI incidence among hospitalized patients with IBD as compared to those without IBD and elderly vs. younger individuals.

The results of this study should be interpreted in the context of its strengths and limitations. It is a population based study without referral, or recall bias and therefore in contrast to studies from tertiary care centers results are more likely to be generalizable to usual clinical practices and recording. However, we did not perform a chart review and have to assume that laboratories are following the legal mandate in the province. In this study, we did not evaluate the impact of misclassification of CDIs.

In conclusion, this study suggests identification of CDIs in hospitals, particularly among individuals with IBD, may not be reliably performed by use of ICD-10 CDI code A04.7 in hospital discharge abstracts.
